# Epigenetics of Skeletal Muscle Atrophy

**DOI:** 10.3390/ijms25158362

**Published:** 2024-07-31

**Authors:** Jiacheng Du, Qian Wu, Eun Ju Bae

**Affiliations:** 1 Department of Biochemistry, Jeonbuk National University Medical School, Jeonju 54896, Republic of Korea; 2 School of Pharmacy and Institute of New Drug Development, Jeonbuk National University, Jeonju 54896, Republic of Korea

**Keywords:** skeletal muscle atrophy, epigenetics

## Abstract

Skeletal muscle atrophy, characterized by diminished muscle strength and mass, arises from various causes, including malnutrition, aging, nerve damage, and disease-related secondary atrophy. Aging markedly escalates the prevalence of sarcopenia. Concurrently, the incidence of muscle atrophy significantly rises among patients with chronic ailments such as heart failure, diabetes, and chronic obstructive pulmonary disease (COPD). Epigenetics plays a pivotal role in skeletal muscle atrophy. Aging elevates methylation levels in the promoter regions of specific genes within muscle tissues. This aberrant methylation is similarly observed in conditions like diabetes, neurological disorders, and cardiovascular diseases. This study aims to explore the relationship between epigenetics and skeletal muscle atrophy, thereby enhancing the understanding of its pathogenesis and uncovering novel therapeutic strategies.

## 1. Introduction

Skeletal muscles, comprising over 600 parts and constituting approximately 40% of body weight, are integral to maintaining body temperature, facilitating physical activity, and regulating energy metabolism. Skeletal muscle atrophy primarily arises from a disparity where protein degradation surpasses synthesis, resulting in a gradual loss of muscle mass and function. Factors contributing to this condition are categorized as congenital, such as amyotrophic lateral sclerosis (ALS), spinal muscular atrophy (SMA), and Duchenne muscular dystrophy (DMD), or acquired, including chronic kidney disease (CKD), diabetes, chronic heart failure, denervation, sarcopenia, and cancer cachexia [1]. During the progression of skeletal muscle atrophy, pathological processes like inflammation, oxidative stress, autophagy, endoplasmic reticulum stress, and mitochondrial dysfunction are pivotal, either independently or collectively exacerbating the condition. This complexity complicates clinical treatment approaches. Current preventive and therapeutic measures encompass exercise, dietary supplements, and medication; nonetheless, an effective treatment remains elusive [2].

Since Conrad Waddington’s initial definition of epigenetics in the early 1940s, interest and research in this field have surged [3]. Significant advancements have been achieved in elucidating epigenetic mechanisms that regulate gene expression. Epigenetics posits that the DNA content in somatic cells remains constant despite environmental changes, with gene expression variations primarily due to chromatin state differences. Beyond chromatin structure, epigenetic inheritance influences gene expression by modulating mRNA transcription and translation. It is now understood that epigenetic modifications, through reversible covalent changes to histones and nucleic acids, regulate chromatin structure and gene expression dynamically, adapting to environmental conditions. The expanding scope of epigenetics now encompasses numerous pathological conditions, including cancer [4,5].

Epigenetic alterations in skeletal muscle manifest with aging, predominantly through increased methylation levels. The methylation status of 200 CpG sites is predictive of the muscle’s chronological age. Additionally, various muscle atrophy models exhibit modifications in histone acetylation and its regulatory factors. Histone acetylases and deacetylases modulate the expression of numerous pathways and genes crucial to skeletal muscle function. Concurrently, non-coding RNAs play a significant role in muscle atrophy [6]. This article aims to offer researchers new insights and directions by focusing on epigenetic modifications and their implications in skeletal muscle atrophy.

## 2. Pathogenesis of Skeletal Muscle Atrophy

**Figure 1:**
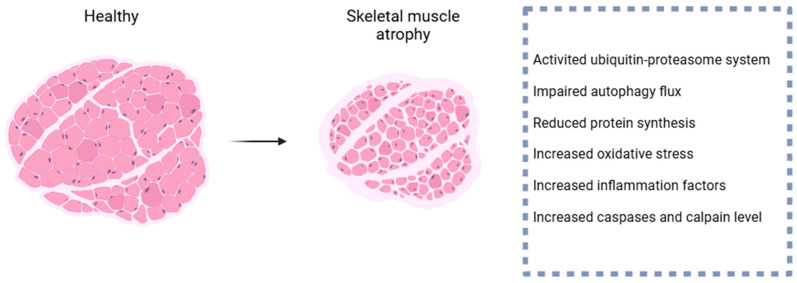
The pathogenesis of skeletal muscle atrophy. Skeletal muscle atrophy is often accompanied by activation of the UPS system, impaired autophagic flux, decreased protein synthesis, and elevated levels of oxidative stress, inflammation, caspases and calpain.

Skeletal muscle atrophy is a debilitating condition lacking targeted treatments and remains poorly understood. Oxidative stress and inflammation significantly contribute to its pathogenesis. The primary sources of reactive nitrogen species and reactive oxygen species in skeletal muscle are mitochondria, xanthine oxidase, uncoupled nitric oxide synthase, and nicotinamide adenine dinucleotide phosphate oxidase [1,7]. Oxidative stress induces damage to proteins, lipids, and DNA within skeletal muscle, leading to protein degradation, cell membrane damage, and apoptosis [7]. Inflammatory factors inhibit protein synthesis and enhance proteolysis via their ligands, thereby exacerbating muscle atrophy. Inflammation also impacts other tissues, such as the digestive system, adipose tissue, and liver, indirectly affecting skeletal muscles [8]. The ubiquitin–proteasome system, autophagy–lysosome system, calpain system, and caspase system are key mechanisms driving skeletal muscle atrophy [9] (Figure 1).

### 2.1. Ubiquitin–Proteasome System

Muscle atrophy F-box/MAFbx (atrogin-1) and muscle ring finger-1 (MuRF-1) are key ubiquitin-E3 ligases involved in skeletal muscle atrophy. MuRF-1 predominantly ubiquitinates myosin heavy chain, myosin light chains 1 and 2, troponin I, and myosin binding protein C [9], whereas atrogin-1 targets eukaryotic translation initiation factor 3 subunit f (eIF3-f), myosin heavy chain, myogenic differentiation antigen (MyoD), and other sarcomeric proteins such as desmin and the intermediate filament protein vimentin [7,10]. Additional ubiquitin-E3 ligases, including ASB2β (associated with mitochondria, contractile proteins, protein synthesis, UPS-mediated protein degradation, and cytoskeleton/sarcomeres) [11], Mindbomb-1 (Mib1, which regulates ubiquitination and proteasome-dependent degradation of Actn3) [12], and NEDD4 (which modulates the ubiquitination of KLF15 and PDLIM7) [13,14], also play critical roles in skeletal muscle atrophy.

### 2.2. Autophagy–Lysosome System

In various types of skeletal muscle atrophy, autophagy flux alterations are common, characterized by an increased LC3BII/LC3B1 ratio and elevated insoluble p62 levels. Studies have demonstrated that in chronic autophagy related 7 (*Atg7)*^−/−^ mice, the proportion of centralized nuclei in extensor digitorum longus (EDL) muscle increases, accompanied by decreased twitch and tetanic force, as well as heightened apoptosis [15]. Furthermore, in sepsis-induced skeletal muscle atrophy, *Atg7* gene knockout (KO) exacerbates muscle atrophy, reduces blood glucose levels, and raises ketone body concentrations [16]. Conversely, Doerr et al. reported that knocking down autophagy related 5 (*Atg5)*, essential for autophagosome formation, significantly improves soleus muscle atrophy and mitigates doxorubicin-induced muscle strength loss in female Sprague Dawley rats [17].

### 2.3. Caspases

Caspases are conserved cysteine proteases vital for cell death and inflammation, primarily encompassing caspases 1–12 and 14 [18]. Caspase-3, which cleaves actomyosin, plays a pivotal role in skeletal muscle atrophy. Elevated caspase activity has been observed in CKD, cancer cachexia, and disuse-induced skeletal muscle atrophy [19,20,21]. In *caspase-3* KO mice, the apoptotic signaling pathway is significantly diminished, leading to notable improvements in denervation-induced muscle atrophy [22]. Additionally, knocking down *caspase-9* significantly enhances the expression of genes associated with cell adhesion, proliferation, growth, development, and division regulation while inhibiting stress response and cell death-related genes [23].

### 2.4. Calpain

Calpains are a family of calcium-activated cysteine proteases that cleave sarcomeres in skeletal muscle, releasing actin and myosin, thus rendering them susceptible to ubiquitination and subsequent degradation [24]. Elevated calpain activity has been observed in conditions such as hydrogen peroxide (H_2_O_2_) exposure, inflammation, diabetes, cachexia, denervation, and disuse-induced skeletal muscle atrophy [25,26,27,28,29].

## 3. The Pathway Related to Skeletal Muscle Atrophy

The primary pathway implicated in skeletal muscle atrophy is the phosphoinositide 3-kinase (PI3K)/serine/threonine-specific protein kinase (AKT) pathway, which regulates the phosphorylation of the mechanistic target of rapamycin kinase (mTOR) and its downstream p70 ribosomal protein S6 kinase (P70S6K) and eIF4E-binding protein 1 (4EBP1), thereby influencing protein synthesis. This pathway also modulates the phosphorylation of forkhead box O (Foxo), promoting the transcription of MuRF-1 and atrogin-1 (ubiquitin-E3 ligase members) and affecting autophagy flux [30]. In patients with pancreatic carcinoma-induced cachexia, AKT protein levels are reduced by 55% compared to non-cachexia patients. Furthermore, the phosphorylation or abundance of Foxo1, Foxo3a, mTOR, and their downstream p70S6K are variably decreased [31]. In patients with sarcopenia, insulin-like growth factor 1 (IGF-1) mRNA levels decrease by 45% and AKT phosphorylation by approximately 30% [32]. Goncalves et al. found that in *AKT1*-KO mice, the weights of the quadriceps, EDL, and gastrocnemius muscles (predominantly type II fibers) are reduced, while *AKT2*-KO leads to reduced EDL and gastrocnemius muscle weights [33]. Additionally, *AKT2*-KO reduces AMPK phosphorylation, myocyte enhancer factor 2A expression in muscle, and mitochondrial DNA (mtDNA) abundance [34].

Pro-inflammatory cytokines such as interleukin-1 (IL-1), tumor necrosis factor-alpha (TNF-α), and interleukin-6 (IL-6) facilitate muscle proteolysis. Studies show elevated IL-6, TNF-α, and C-reactive protein (CRP) levels correlate with diminished grip strength and muscle mass [35,36]. The nuclear factor kappa-light-chain-enhancer of the activated B cell (NF-κB) pathway, related to inflammation, also significantly contributes to skeletal muscle atrophy, particularly in cancer cachexia, sarcopenia, and inflammation-induced muscle atrophy [37,38,39,40]. For instance, NF-κB pathway activity in the muscles of 70-year-olds is approximately four times higher than in young adults [40], and in patients with gastric cancer, phospho-p65 increases by approximately 25% while IκBα expression decreases by about 25% [41]. Nuclear factor kappa B subunit 1 (*Nfkb1*) KO notably reduces disuse-induced skeletal muscle atrophy in mice, primarily by enhancing the muscle fiber area of fast fibers [42].

The AMPK pathway is essential in skeletal muscle atrophy, as it can inhibit protein synthesis by modulating the phosphorylation of mTOR and eukaryotic initiation factor 2 [43]. Additionally, AMPK promotes mitochondrial biosynthesis and reduces oxidative stress through SIRT1 and PGC1-α [44]. In patients with non-small cell lung cancer, levels of various AMPK subunits are elevated, likely due to AMPK’s protective activation, as suggested by Raun [45]. *AMPKα1/α2* KO in mice leads to reduced body weight and muscle fibers, impairing muscle function [46]. Conversely, Guo et al. found that *AMPKα2* KO reduces protein degradation and mitigates denervation-induced muscle atrophy, indicating model-specific effects [47].

**Figure 2:**
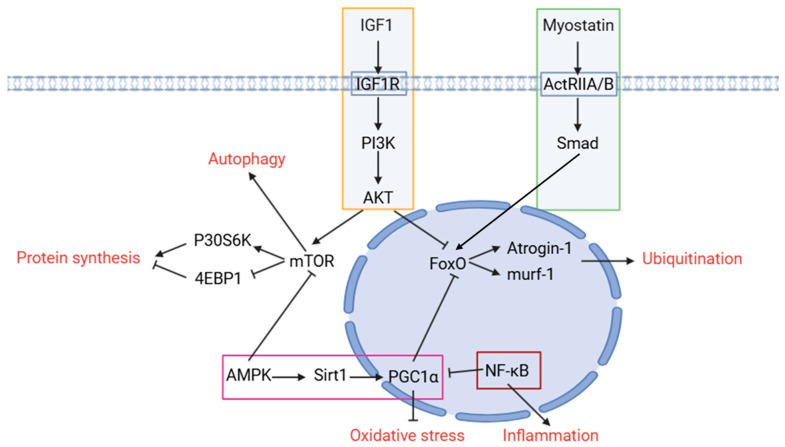
The pathway related to skeletal muscle atrophy. PI3K/AKT, NF-κB, AMPK and myostatin pathways play an important role in muscular atrophy. The PI3K/AKT pathway can promote protein synthesis, inhibit the UPS system and improve autophagy flux. The NF-κB pathway can promote inflammation and the UPS system. AMPK is more responsible, it can inhibit mTOR but promote PGC1α. Myostatin can affect the UPS system through the Smad pathway.

Myostatin, also known as growth differentiation factor-8 (GDF-8), is a member of the TGFβ family that negatively regulates skeletal muscle growth and development [48]. It induces muscle atrophy by affecting the expression of ubiquitin-E3 ligases (MuRF-1 and atrogin-1) via the Smad pathway [49]. Elevated myostatin levels have been observed in patients with gastric and lung cancers [50,51]. Higher serum myostatin levels are also found in patients with hepatocellular carcinoma compared to healthy individuals, correlating with a worse prognosis [52,53]. In the elderly with sarcopenia, muscle and serum myostatin levels are increased compared to younger individuals [54,55]. In patients with inflammatory bowel disease, serum myostatin levels correlate with sarcopenia severity [56]. *Myostatin* KO has been shown to improve muscle atrophy caused by glucocorticoids and aging [57,58], while its overexpression negates muscle hypertrophy induced by IGF-1 overexpression [59]. However, *myostatin*^−/−^ animals, despite having greater muscle volume and body weight, exhibit reduced specific force and fatigue resistance compared to wild-type animals [60] (Figure 2).

## 4. Epigenetics in Different Kinds of Skeletal Muscle Atrophy

Skeletal muscle atrophy, characterized by disrupted metabolic homeostasis, often involves redox adaptation and mitochondrial dysfunction, leading to metabolic reprogramming. These metabolic disorders are intricately linked to epigenetics and encompass DNA modifications, histone modifications, chromatin remodeling, and RNA epigenetics [61,62]. This section will explore the roles of various epigenetic modifications in skeletal muscle atrophy.

### 4.1. DNA Modification

#### 4.1.1. The Role of DNA Modification in Skeletal Muscle Atrophy

At least 17 chemical modifications have been identified in DNA, playing key roles in various biological processes and diseases, including development, aging, and cancer. In mammalian genomes, the most prevalent DNA modification is the methylation at the fifth carbon of cytosine (5-methylcytosine or 5mC), with 70 to 80% of CpGs being methylated. Other notable DNA modifications in mammals include 5-hydroxymethylcytosine (5hmC), 5-carboxylcytosine (5caC), and 5-formylcytosine (5fC) [63]. 

In age-matched patients with sarcopenia, whole blood samples revealed that the methylation levels of CTSB_15, CXCL12_22, and FGF2_30 were lower than those of the normal group, while CTSB_17 and FGF19_28 exhibited higher methylation levels. Notably, FGF2_30 methylation positively correlated with appendicular skeletal muscle index, grip strength, and gait speed [64]. A weighted sum of sarcopenia-driven CpG-site methylation levels was significantly elevated in patients with sarcopenia and negatively correlated with vastus lateralis anatomical cross-sectional area, maximum isometric elbow flexion, and knee extension [65]. Kyoto encyclopedia of genes and genomes (KEGG) analysis indicated that genes related to methylation differences in sarcopenia were mainly enriched in pathways involving actin cytoskeleton regulation, muscle function, and energy metabolism [66].

In the longissimus dorsi muscle of 7-year-old pigs, methylated DNA immunoprecipitation sequencing revealed a non-significant decrease in 5mC levels compared to half-year-old pigs. However, DNA methyltransferase (DNMT)3b mRNA levels significantly increased. A correlation analysis of differentially methylated region (DMR)-mRNA pairs identified 9234 differentially methylated regions between these groups. Hypomethylated gene body regions were significantly enriched in processes related to protein catabolism, energy metabolism, and proteolysis. Additionally, increased Foxo3 mRNA levels and decreased methylation of its gene body were observed in the skeletal muscle of 7-year-old pigs, while FGFR1, a gene inhibiting muscle atrophy, showed downregulated expression and hypermethylation [67].

In tetrodotoxin-induced skeletal muscle atrophy, murf-1 and atrogin-1 exhibited reduced DNA methylation and increased mRNA levels. Post-exercise, murf-1 DNA methylation significantly increased [68]. In the soleus muscle of mice, one week post-immobilization, the nNOS gene methylation level significantly rose [69]. Conversely, UBR5, an E3 ubiquitinase, showed reduced methylation in disused muscle atrophy, with exercise promoting its methylation level [70].

#### 4.1.2. The Protective Role of DNMT on Skeletal Muscle Atrophy

DNA methyltransferases primarily include DNMT1, DNMT3A, and DNMT3B. DNMT3A and DNMT3B catalyze de novo DNA methylation, while DNMT1 functions as a processive DNA methyltransferase. Key DNA demethylases belong to the TET family, including TET1, TET2, and TET3. TET1 mediates the conversion of 5mC to 5hmC, with the latter two acting as intermediates in active DNA demethylation pathways [63].

In denervation-induced skeletal muscle atrophy, DNMT3A expression is significantly reduced, resulting in hypomethylation of the Gdf5 and Fn14 promoter regions. This hypomethylation impairs cell differentiation and disrupts the NF-κB and UPS, contributing to muscle atrophy [71,72].

### 4.2. Histone Modifications

Histones are basic proteins in the nuclei of eukaryotic cells that bind to DNA, forming nucleosomes together with 147 DNA base pairs. Each nucleosome is an octamer composed of two copies each of histones H2A, H2B, H3, and H4. Amino acid sequences of histones are conserved across species. Post-translational modifications of histones, including acylation (e.g., acetylation, butyrylation, benzoylation, glutarylation, crotonylation, lactylation), ADP-ribosylation, glycosylation, dopaminylation, methylation, serotonylation, phosphorylation, threosylation, and ubiquitination, play pivotal roles in chromatin condensation, gene transcription regulation, and DNA replication [73,74,75].

#### 4.2.1. Histone Acetylation

Histone acetylation involves the binding of an acetyl group to a lysine residue at the N-terminus of histones. This process is mediated by “writing” enzymes known as histone acetyltransferases (HATs), “erasing” enzymes called histone deacetylases (HDACs), and “reading” enzymes containing bromodomains that recognize and bind to acetylated lysine residues. Histone acetylation disrupts protein-DNA interactions and loosens chromatin structure, thereby promoting gene transcription [76,77,78].

##### The Role of Histone Acetylation in Skeletal Muscle Atrophy

Histone acetylation plays a vital role in normal muscle function by regulating energy metabolism and glucose utilization in muscle cells. For instance, long-term exercise significantly increases histone H3 acetylation [79]. Ryder et al., using a peptide IP proteomic approach with an anti-acetyl-lysine antibody or a ubiquitin residue motif antibody followed by mass spectrometry, confirmed that histone acetylation increases while ubiquitination decreases in rat skeletal muscle from a cast immobilization-induced atrophy model. This change correlates with the increased transcription of genes related to muscle atrophy. Conversely, proteins associated with muscle contractions, such as alpha-actin, troponin C, myosin light chains 3 (MLC3), myosin heavy chain I, troponin T, and myosin heavy chain IIa, are deacetylated and ubiquitinated [80]. Furthermore, Kawano et al. demonstrated that in denervation-induced skeletal muscle atrophy, histone acetylation of fast and slow muscle fiber-related genes significantly decreased in EDL muscles [81]. Additionally, in aging mice, the gastrocnemius muscle weight-to-body weight ratio significantly declined in 12- and 24-month-old mice, while atrogin-1 mRNA levels increased. Acetylation of histone H3, H3K9, and H3K27 negatively correlated with age and was significantly reduced in older mice [82]. 

##### HATs Play Different Roles in Different Types of Muscular Atrophy

HATs are primarily classified into three families: GNAT, MYST, and CBP/p300. The GNAT family includes GCN5, PCAF, HAT1, Elp3, and Hpa2. The MYST family comprises Tip60 (HAT6), Sas2, Sas3, Esa1, MOF, MORF, and Hbo1. HATs facilitate DNA repair and its dissociation from the histone octamer [83].

Studies indicate that the expression of p300, Cbp, Pcaf, and Moz is elevated in immobilization, denervation, and starvation-induced muscle atrophy models, while Gcn5 expression increases only in the former two [84]. Muscle-specific knockout of *Gcn5* results in increased dystrophin expression, leading to myopathy [85]. Conversely, Lee et al., demonstrated that *GCN5* knockdown ameliorates starvation-induced muscle atrophy by altering p65K310 acetylation [86].

Regarding CBP/p300, activation of p300 is increased in dexamethasone, palmitic acid, or sepsis-induced skeletal muscle atrophy [87,88,89]. Knockdown of *p300* improves muscle condition by modulating the acetylation of p65, FOXO1, and FOXO3a or by affecting autophagic flux [87,88,90]. Researches by Sin et al. and Liang et al. revealed that p38β MAPK phosphorylates p300 at serine-12, thereby stimulating C/EBPβ acetylation and contributing to cancer cachexia [91,92].

##### HDACs Play Different Roles in Congenital and Acquired Muscular Atrophy

HDACs are enzymes that remove acetyl groups from histones, regulating chromatin structure and gene expression. The HDAC family includes a large group of proteins classified into five groups: class I (HDAC 1, 2, 3, 8), class IIa (HDAC 4, 5, 7, 9), class IIb (HDAC 6, 10), class III (Sirtuins), and class IV (HDAC 11), all highly conserved across species. HDACs play critical regulatory roles in various biological processes, and their abnormal activity is associated with diseases such as neurodegenerative disorders and cancers. Research on HDACs in skeletal muscle mainly focuses on HDAC1, 4, 6, and Sirtuins [93].

Beharry et al. found that HDAC2, 4, 6, and Sirtuin1 levels significantly increased in skeletal muscle atrophy induced by denervation, immobilization, and nutrient deprivation, while HDAC1 and 3 increased in the first two models. Conversely, HDAC7 was significantly reduced in all three models [84]. Moresi et al. demonstrated that knockout of *HDAC4* or HDAC5, individually or simultaneously, increased TA muscle weight compared to controls and significantly decreased muscle atrophy markers (MuRF-1 and atrogin-1) in denervation-induced atrophy model mice [94]. Additionally, *HDAC4* gene KO improved autophagic flux and oxidative stress inhibition caused by denervation and promoted MHC expression by enhancing its acetylation [95]. However, in an ALS model, *HDAC4 KO* led to earlier ALS onset with reduced body weight, TA muscle cross-section area, and NMJ surface area compared to controls [96]. HDAC1 regulates E3 ubiquitin ligase and autophagy by modulating Foxo1a phosphorylation, and its inhibition significantly improves immobilization-induced skeletal muscle atrophy [97]. In denervation-induced muscle atrophy, HDAC6 is regulated by Foxo3a and interacts with atrogin-1 to promote MyoD ubiquitination and degradation, thus promoting muscle atrophy [98].

Several HDAC inhibitors, such as trichostatin A, butyrate, MS-275, LMK-235, NVS-HD1, tubastatin A, valproic acid, and HC toxin, have shown efficacy in various muscle wasting models (dexamethasone, sarcopenia, unloading, denervation, starvation, DMD, cancer cachexia, cigarette smoke exposure, ALS, Table 1) [87,95,97,99,100,101,102,103,104,105,106,107]. For example, in SOD1-G93A mice, a model of ALS, histone H3 acetylation was significantly reduced. Trichostatin A, an HDAC inhibitor, significantly improved histone H3 acetylation, inhibited motor neuron death and axonal degeneration, and enhanced motor function and muscle condition. The drug also ameliorated skeletal muscle atrophy caused by posterior suspension.

#### 4.2.2. Histone Methylation

##### The Role of Histone Methylation in Skeletal Muscle Atrophy

Histone methylation involves the addition of methyl groups to lysine or arginine residues of histones, facilitated by histone methyltransferase (HMT). This modification can occur at various positions on lysine and arginine, resulting in monomethylation (me1), dimethylation (me2), or trimethylation (me3). Primarily targeting histones H3 and H4, histone methylation significantly influences gene expression, depending on the specific residue and methylation degree. For instance, H3K4me3, the trimethylation of lysine 4 on the H3 histone, typically occurs in promoter regions and correlates with active gene transcription. Conversely, H3K9me3, the trimethylation of lysine 9 on the H3 histone, is linked to gene silencing and heterochromatin formation. Similarly, H3K27me3, the trimethylation of lysine 27 on H3, is generally associated with gene silencing [108,109,110]. Histone methylation is involved in various pathophysiological processes, including gene expression regulation, cell differentiation and development, and the maintenance of genome stability and integrity [111]. Studies reveal that in rat gastrocnemius muscle, the H3K9me3 histone methylation modification decreases with age, showing a significant negative correlation with atrogin-1 mRNA levels and a positive correlation with relative muscle weight [82]. 

##### HMTs Protects Skeletal Muscle from Atrophy

In the advanced stage of denervation-induced skeletal muscle atrophy, the expression of protein arginine methyltransferases (PRMT) 1, CARM1 (also known as PRMT4) and PRMT5 are elevated [112]. PRMT1, an N-arginine methyltransferase, mediates the transfer of methyl groups from S-adenosyl-L-methionine (SAM or AdoMet) to the guanidine termini on arginine residues’ side chains, thus facilitating the formation of dimethylarginine [113]. This enzyme plays a key role in histone methylation and significantly impacts skeletal muscle atrophy, with research indicating that *PRMT1* KO induces muscle atrophy. Additionally, PRMT1 associates with the promoter region of PRMT6, and its gene KO enhances PRMT6 expression, excessively activating Foxo3 and promoting muscle atrophy [114]. Similarly, CARM1 is pivotal in muscle atrophy, with its KO disrupting mitophagy and autophagy, leading to muscle degradation [115]. Stouth et al. demonstrated that *CARM1* deficiency only reduced the exercise capacity and endurance in male mice without affecting muscle mass or denervation-induced atrophy [116].

Unlike other methyltransferases, DOT1L lacks the Su(var)3-9, enhancer of zeste, and Trithorax (SET) domain and exclusively methylates lysine [117]. Research has shown that histone methyltransferase DOT1L expression is reduced in the vastus lateralis muscle of COPD individuals with sarcopenia, and in vitro DOT1L knockdown significantly upregulates p21 expression [118].

#### 4.2.3. γ-H2AX (a Type of Histone Phosphorylation) Is Expressed More Frequently in Skeletal Muscle Atrophy

Nearly all types of histones undergo phosphorylation at specific residues, a modification essential for transcriptional regulation, cell division, DNA damage repair, hetero-chromatin formation, and gene silencing. For instance, the phosphorylation of the third serine residue on H3 histone (H3S10) is associated with chromosome condensation, essential for chromosome segregation during cell division. Notably, DNA damage repair is significantly marked by the phosphorylation of H2A histone family member X (H2AX) at serine 139, a key indicator of aging [119,120].

Histone phosphorylation, especially of γ-H2AX, also plays a vital role in skeletal muscle atrophy. Studies have shown that in primary human muscle progenitor cells isolated from the lateral thigh and cultured for replication and senescence, γ-H2AX expression markedly increases compared to normal cells. This increase is accompanied by a significant reduction in the expression of cell differentiation markers (Myog, MyHC, MyoD), reduced fusion, decreased myotube area, and elevated TGF-β levels [121]. Additionally, skeletal muscle atrophy associated with chronic kidney disease (CKD) and spinal muscular atrophy (SMA) is linked to heightened γ-H2AX expression [122,123]. During SYUIQ-5-induced cellular senescence, γ-H2AX expression shows a negative correlation with MyHC expression and a positive correlation with muscle atrophy markers (murf-1 and atrogin-1) [124].

#### 4.2.4. Histone Ubiquitination Reduced during Skeletal Muscle Atrophy

Histone ubiquitination is a key post-transcriptional modification process wherein small ubiquitin proteins are covalently attached to specific amino acid residues of histones. This modification can alter chromatin structure, thereby influencing transcription factor binding and gene activity. As a reversible process, ubiquitination allows for the removal of ubiquitin molecules at various intervals. Ubiquitin can attach to multiple lysine residues on histones, forming polyubiquitin chains. The varying lengths (monoubiquitination, polyubiquitination) and types of ubiquitin chains convey distinct biological information and correspond to different degradation pathways and functional outcomes. Histone ubiquitination is implicated in numerous biological processes, such as protein degradation, DNA repair, cell cycle regulation, gene silencing, and chromatin remodeling [125]. In a rat model of immobilization-induced skeletal muscle atrophy, histone ubiquitination was significantly reduced, correlating with a marked decrease in soleus muscle mass compared to the control group, and an increase in the ubiquitination of muscle contraction proteins [80].

#### 4.2.5. Histone Lactylation May Play a Critical Role in Skeletal Muscle Atrophy

Histone lactylation is an epigenetic modification wherein lactate molecules are added to specific amino acid residues of histones, playing a significant role in cell metabolism and gene expression regulation. This modification predominantly occurs on the lysine residues of histones H3 and H4 and is mediated by enzymes such as lactation modifying enzyme (LME). Lactate, serving as a precursor for histone lysine lactylation (Kla), stimulates gene transcription within chromatin. Histone lactylation can influence the structure and function of histones, thus altering chromatin architecture and gene expression. This modification is implicated in various biological processes, including cell proliferation, energy metabolism, differentiation, and immune response. As the primary product of glycolysis, particularly under hypoxic conditions (e.g., during intense muscle exercise), lactic acid rapidly supplies energy to muscles [126]. Additionally, lactic acid functions as a vital signaling molecule, impacting several pathological and physiological processes such as intracellular reactive oxygen species, mitochondrial biogenesis, and fatty acid oxidation. It is currently posited that P300, HDAC1-3, and sirtuin1-3 enzymes can establish and remove histone lactate [127,128]. These genes have demonstrated significant roles in various models of skeletal muscle atrophy [62,88,129,130,131].

### 4.3. RNA Modification

RNA modification involves chemical alterations to RNA molecules post-transcription, significantly impacting their function and stability. This process is both complex and diverse, encompassing over 170 types of chemical modifications beyond the well-known m6A modification. These modifications are prevalent in both noncoding RNA and messenger RNA, playing pivotal roles in RNA stability, translation efficiency, and intracellular localization. As a key component of epigenetics, RNA modification intricately regulates gene expression by finely tuning RNA structure and function. Together, these chemical modifications form a sophisticated regulatory network essential for maintaining cellular physiological functions and contributing to the onset and progression of various diseases [132,133].

#### 4.3.1. RNA Methylation

##### The Role of RNA Methylation in Skeletal Muscle Atrophy

In 1974, methylation was first identified in mRNA, with the modified adenine known as m6A being the most common post-transcriptional modification in mRNA. Typically, 1–2 m6A residues are found per 1000 nucleotides. RNA methylation plays a critical role in gene expression regulation, with N6-adenosine methylation (m6A) being the predominant chemical modification on mRNA. The m6A modification involves the addition of a methyl group at the N6 position on the adenosine residue of mRNA molecules, catalyzed by specific enzymes. This chemical modification is widespread in eukaryotes and significantly impacts mRNA processing, stability, and translation. M6A modification regulates mRNA precursor splicing and RNA nuclear export and influences mRNA translation and stability. It also promotes circular RNA translation and contributes to cell differentiation and tumor development. The dynamic and reversible nature of m6A modification involves methyltransferases (writers), demethylases (erasers), and methyl-binding proteins (readers). Methyltransferases such as methyltransferase like (METTL) 3 and METTL14 catalyze the m6A modification, while demethylases like fat mass and obesity-associated (FTO) and alpha-ketoglutarate-dependent dioxygenase alkB homolog 5 (ALKBH5) remove these methyl groups, allowing for flexible gene expression regulation [134,135].

M6A methylation of mRNA has also been observed in the skeletal muscles of various species, potentially influencing skeletal muscle differentiation. During prenatal porcine skeletal muscle development, m6A predominantly accumulates in the coding sequence and three prime untranslated region (3′-UTR), enriched in processes related to RNA binding, nucleocytoplasmic transport, and macromolecular metabolism. The m6A and mRNA levels of certain genes involved in skeletal muscle differentiation, such as MYH2 (which encodes MyHC), are significantly elevated [136]. Additionally, Gheller et al. found that m6A levels increase during the early stages of skeletal muscle regeneration but decrease during C2C12 differentiation, with m6A primarily concentrated in the coding region and 3′-UTR [137].

##### The Role of Methyltransferases and Demethylases in Skeletal Muscle Atrophy

Knocking down the methyltransferase *METTL14* in C2C12 cells inhibits differentiation, as evidenced by decreased levels of differentiation markers MyHC, MyoD, and MyoG while promoting cell growth. Conversely, METTL3, another methyltransferase, is reduced in myoblasts. Unlike METTL14, *METTL3* knockdown enhances skeletal muscle cell differentiation and inhibits proliferation [137]. 

In denervation-induced skeletal muscle atrophy, numerous m6A-modified RNAs are predominantly downregulated. These modifications are enriched in functions such as ubiquitin–protein ligase activity, ATP binding, zinc ion binding, transcription coactivator activity, protein phosphatase binding, RNA polymerase II cis-regulatory region sequence-specific DNA binding, DNA binding, glucocorticoid receptor binding, nucleic acid binding, and mannosyl-oligosaccharide 1,2-alpha-mannosidase activity. During this process, demethylases like FTO and ALKBH5 significantly increase. Furthermore, 3-Dezidenosine (DAA) in mouse muscle promotes ALKBH5 and FTO expression, substantially reducing m6A levels in muscles and leading to decreased muscle weight and cross-sectional area. Local injection of R-2-HydroxyglutaraTe (R-2HG) in denervation-induced skeletal muscle atrophy inhibits FTO and ALKBH5 expression, promotes m6A modification, and ameliorates muscle atrophy [138]. Liu et al. also confirmed that m6A levels decrease while ALKBH5 expression increases in the gastrocnemius muscle of denervation-induced atrophy models, with ALKBH5 targeting HDAC4 to enhance Foxo3 expression and muscle atrophy [139].

#### 4.3.2. Other Kinds of RNA Modification

In addition to RNA methylation, other types of RNA modifications include ac4C, 2-thiouridine (s2U), A-I (adenosine to inosine) RNA editing, pseudouridine (Ψ), and the 5′-cap (m7GpppN). A-I RNA editing is a post-transcriptional modification process where adenosine is converted into inosine, catalyzed by specific adenosine deaminases acting on RNA (ADARs). This modification can alter RNA structure and function by changing encoded amino acids, affecting splicing sites, or regulating RNA stability and translation efficiency. A-I RNA editing is critically involved in various biological processes, including neuronal development, immune response, and viral infection. Defective RNA editing has been linked to numerous diseases, such as neurodegenerative disorders, cancer, and autoimmune diseases [133].

ADAR2 is an enzyme responsible for A-to-I editing in RNA molecules, highly expressed in the mammalian nervous system and significantly influencing neuronal function and gene regulation. ADAR2’s functions include regulating A-to-I RNA editing, RNA splicing, and affecting RNA stability and translation efficiency. Research has shown that *ADAR2* KO can modulate high-fat diet-induced skeletal muscle atrophy via the AKT/Foxo1 pathway and inflammation. Specifically, *ADAR2* gene KO promotes the phosphorylation of AKT and Foxo1 while inhibiting M1 and M2 macrophage markers (iNOS, IL-12, Arg1, Fizz1, Ym1, and IL-10) [140].

### 4.4. Noncoding RNA

Noncoding RNA (ncRNA) refers to RNA molecules that do not encode proteins but regulate gene expression and other biological processes. NcRNA is classified by length and function into small noncoding RNA (<200 nt) and long noncoding RNA (lncRNA) (>200 nt). Small noncoding RNA includes: (1) microRNA (miRNA): approximately 22 nucleotides long, miRNA regulates gene expression by binding to the 3′ untranslated region (3′UTR) of mRNA, inducing mRNA degradation or inhibiting translation. miRNA plays a critical role in development, cell proliferation, differentiation, and apoptosis; (2) small interfering RNA (siRNA): ranging from 21–23 nucleotides in length, siRNA uses the RNA interference (RNAi) pathway to perfectly complement target mRNA, causing its degradation and thus inhibiting gene expression. siRNA is primarily used to study gene function and treat specific diseases; (3) PIWI-interacting RNA (piRNA): spanning 24–31 nucleotides, piRNA is mainly expressed in germ cells and, by binding to Piwi proteins, inhibits transposon activity, protecting genome integrity. LncRNA has multiple regulatory functions, including gene transcription regulation, RNA splicing, chromatin structure modification, acting as molecular scaffolds, and modulating protein-RNA interactions. Small nucleolar RNA (snoRNA) primarily functions within the nucleus. Circular RNA (circRNA) is a closed circular RNA molecule found in eukaryotic cells, which can regulate gene expression at the transcriptional and post-transcriptional levels, such as acting as sponges for miRNA and RNA-binding proteins [141,142].

#### 4.4.1. miRNA

miRNA is a small non-coding RNA molecule, approximately 22 nucleotides in length, that regulates gene expression by complementing the 3′ untranslated region (3′UTR) of target mRNA. miRNA is pivotal in various biological processes, including cell proliferation, differentiation, apoptosis, and development. The biosynthesis of miRNA involves several steps: transcription (producing primary miRNA (pri-miRNA) through RNA polymerase II transcription), splicing (processing precursor miRNA of about 70 nucleotides by the Drosha-DGCR8 complex), nuclear export (transporting from the nucleus to the cytoplasm via Exportin-5), further processing (Dicer enzyme cleaves pre-miRNA into double-stranded RNA, selecting one strand as mature miRNA (guide strand) while the other (passenger strand) is typically degraded), and forming RISC complexes (binding with Argonaute protein to create RNA-induced silencing complexes (RISC)). The RISC complex mediates gene silencing by guiding miRNA to bind to target mRNA. Various miRNAs have been shown to play vital roles in different types of skeletal muscle atrophy.

Gagan et al. demonstrated that MyoD can bind to the miR-378 gene, leading to transcriptional activation and chromatin remodeling, thus promoting miR-378 expression. MiR-378 further enhances MyoD expression by inhibiting MyoR [143]. Additionally, miR-494-3p can influence the expression of P300 and its downstream targets, MYOD and MYH2 [144].

In denervation-induced skeletal muscle atrophy, miR-142a-5p exacerbates the condition by binding to the 3′UTR of MFN1 mRNA, inhibiting its expression [145]. Overexpression of miR-29 and miR-125b-5p can ameliorate denervation and fasting-induced skeletal muscle atrophy by targeting murf-1 and TRAF6, respectively [146]. Conversely, overexpression of miR-322 and miR-542 in skeletal muscle significantly contributes to atrophy by binding to the 3′UTR of eIF4B, eIF2B5, eIF4E, eIF4G1, and eIF3M, inhibiting their expression [147]. 

Studies have demonstrated that aging-induced atrophic myotubes and macrophages release exosomes enriched with miR-690, which inhibits the differentiation of C2C12 and satellite cells by regulating Mef2a, Mef2c, and Mef2d expression [148]. Overexpression of miR-181a enhances mitochondrial function in aged mice by downregulating p62, Park2, and DJ-1 [149]. Soriano et al. reported that miR-181a decreases the diameter of C2C12 myotubes by inhibiting sirtuin1 expression [150]. In aged mice, the overexpression of miR-434-3p in myotubes reduces the activation of caspase-8, caspase-3, and caspase-9 by inhibiting eIF5A1 [151]. In aged rats, miR-29 plays distinct roles in kidney disease and diabetes-induced skeletal muscle atrophy. Hu et al. confirmed a significant increase in miR-29 expression in aged mice and rats, potentially mediated by Wnt-3a. MiR-29 inhibits the translation of myoblast proliferation mediators by binding to the 3′UTR of IGF-1, p85a, and B-myb, promoting aging-related indicators and leading to skeletal muscle atrophy [152]. In a zebrafish sarcopenia model, miR-128 expression is elevated, and aerobic exercise inhibits its expression, likely due to miR-128 targeting the 3′UTR of IGF-1 [153]. The NF-κB subunit p50 binds to the promoter region of miR-532-3p, and miR-532-3p binds to the 3′UTR of BAK1. Therefore, in skeletal muscle atrophy, p50 inhibits miR-532-3p expression, thereby promoting BAK1 expression and cell apoptosis [154].

In dexamethasone-induced skeletal muscle atrophy, overexpression of miR-486 and miR-182 ameliorates atrophy by reducing protein levels of Foxo1 and Foxo3, respectively [155,156]. MiR-322 targets 3′UTR inhibitors of IGF-1R and INSR, exacerbating dexamethasone-induced muscle atrophy [157]. MiR-320 binds to the 3′UTR of growth factor receptor-binding protein-2 (Grb2), mitigating dexamethasone-induced muscle atrophy [158]. Inhibition of miR-29b ameliorates dexamethasone-, denervation-, H_2_O_2_-, and TNF-α-induced muscle atrophy by regulating IGF-1 and PI3K (p85α) expression [159]. MiR-23a binds to the 3′UTR of murf-1 and atrogin-1, improving dexamethasone-induced muscle atrophy [160]. MiR-27b-3p ameliorates dexamethasone-induced atrophy by inhibiting Cbl-b expression [161].

MiR-29c ameliorates LLC-induced skeletal muscle atrophy by targeting LIF [162]. Tunicamycin-induced oral squamous cell carcinoma (OSCC) cell-conditioned medium contains exosomes enriched with miR-181a-3p, which induces skeletal muscle atrophy [163]. MiR-18a binds to the 3′UTR of IGF1, inhibiting its expression and leading to skeletal muscle atrophy [164]. Exosomes derived from mesenchymal stem cells are rich in miR-145-5p, which can improve busulfan and cyclophosphamide-induced skeletal muscle atrophy by targeting the 3′UTR of ACVR2A and ACVR1B [165].

In unilateral ureteral obstruction mouse models or CKD mice, the expression of miR-29 and miR-26a is decreased [166,167,168,169]. Overexpression of miR-27 and miR-23 ameliorates CKD-induced skeletal muscle atrophy by negatively regulating PTEN, caspase-7, and FoxO1 [19]. Additionally, miR-27a binds to myostatin [170]. Downregulation of miR-29 increases YY1 expression, leading to skeletal muscle atrophy [167]. Conversely, overexpression of miR-29b in C2C12 significantly promotes the expression of muscle atrophy-related genes and reduces muscle fiber diameter [159]. Local intramuscular injection of miR-29-loaded exosomes improves skeletal muscle atrophy and renal fibrosis in mouse models [169]. Overexpression of miR-486 in CKD mice ameliorates skeletal muscle atrophy by affecting FoxO1 and PTEN [171].

In the diabetes-induced skeletal muscle atrophy model, the expression of miR-182 and miR-23a is reduced [156,172]. Local injection of AAV-miR-23a/27a into the tibialis anterior muscle promotes phosphorylation of AKT and SMAD2/3 and inhibits myostatin expression, thus improving diabetes-induced skeletal muscle atrophy [173]. Studies have shown that miR-193b expression is increased in the serum of patients with type 2 diabetes. Inhibition of miR-193b improves muscle loss and dysfunction in db/db mice by targeting PDK1 and promoting the AKT/mTOR pathway [174].

SMN binds to the upstream genomic regions of miR-206 and miR-1 [175]. A tail vein injection of AAV-miR-298 effectively ameliorates the phenotype of mice with bulbar and SMA [176]. Following spinal cord injury, the expression of miR-499-5p and miR-208b decreases, with miR-208b targeting and binding to myostatin to reduce its expression [177].

In skeletal muscle atrophy induced by hindlimb suspension, the expression of miR-203a-3p, miR-499, and miR-6516 precursors is significantly downregulated, while the miR-206 precursor is increased [178,179,180]. Among these, miR-499 regulates MyHC expression by binding to the 3′UTR of Sox6, leading to skeletal muscle atrophy [179]. Local injection of miR-6516 into the TA muscle improves skeletal muscle atrophy caused by hindlimb immobilization [180].

Under inflammatory conditions, miR-140 negatively regulates the Wnt pathway, HEY1, and Notch 1, resulting in skeletal muscle atrophy [181]. However, Shin et al., demonstrated that knocking out the miR-140 gene does not exacerbate LPS-induced skeletal muscle atrophy [182]. Inhibiting miR-21 significantly improves myotube diameter in myosatellite cells [183]. Overexpression of miR-497-5p in C2C12 cells induces muscle atrophy by regulating the insulin signaling pathway [184]; while miR-1290 promotes skeletal muscle differentiation by targeting Foxo3a and inhibiting the expression of murf-1 and atrogin-1 [185].

Compared to controls, the expression of miR-424-5p, miR-206, miR-1, miR-27a, miR-542-3p/5p, and miR-145-5p is upregulated in muscle biopsies of patients with COPD [186,187,188,189,190]. However, miR-1 is significantly decreased in the quadriceps of patients with COPD [191]. MiR-206 expression is elevated in the muscles of G93A-SOD1 transgenic mice, SMAII mice, and denervation-induced skeletal muscle atrophy model mice, while miR-133a expression is decreased in G93A-SOD1 transgenic mice [192]. In the gastrocnemius muscle of mdx4cv mice, there is a significant decrease in miR-30 family expression [193]. MiR-1/206/133 induces neuromuscular symptoms and skeletal muscle atrophy in mice by binding to the 3′UTR of CRK [194].

#### 4.4.2. LncRNA

LncRNA is a type of RNA molecule longer than 200 nucleotides that does not encode proteins. Despite not encoding proteins directly, lncRNA plays pivotal regulatory roles in various biological processes, including gene expression regulation, chromatin structure remodeling, RNA processing, and protein interaction. LncRNA is involved in cell differentiation and development, gene imprinting regulation, and numerous disease processes.

In skeletal muscle, lnc-MYH regulates the composition of the INO80 chromatin remodeling complex. Knockout of *lnc-MYH* modulates the proliferation of MuSCs cells via INO80, resulting in muscle hypertrophy [195]. During muscle satellite cell differentiation, lncMREF interacts with Smarca5 to enhance chromatin accessibility and the binding of the p300/CBP/H3K27ac complex, thereby promoting MyoD expression [196]. Cabianca et al. demonstrated that in facioscapulohumeral muscular dystrophy, the elevated expression of DBE-T (a lncRNA) induces FSHD by recruiting Ash1L (a trithorax group protein) to the FSHD locus, driving histone H3 lysine 36 dimethylation, chromatin remodeling, and 4q35 gene transcription [197]. Additionally, ChRO1, a lncRNA expressed during myoblast differentiation, influences DNA methylation and chromatin compaction in pericentromeric/centromeric regions [198]. Dum binds to the Dppa2 promoter region and recruits Dnmts, promoting DNA methylation of the Dppa2 promoter [199]. DNA methylation also affects lncRNA expression; the methylation level of the GTL2 gene increases with age [200], while ALKBH5 regulates the m6A demethylation of MYH1G-AS [201].

Compared to the control group, the expression of PRKG1-AS1, lnc-MALAT1, and Chronos is upregulated, whereas GPRC5D-AS1 and AC103740.1 are downregulated in the muscles of older individuals [202,203,204]. GPRC5D-AS1, acting as a competing endogenous RNAs (ceRNA) of miR-520d-5p, inhibits the regulation of muscle regulatory factors and cell apoptosis [205]. Inhibition of Chronos leads to muscle hypertrophy through the regulation of Bmp7 [204].

In dexamethasone-induced skeletal muscle atrophy, the expression of Atrolnc-1, Dum, MAR1, lnc-MD1, and SYISL increases, while Myolinc decreases [206,207]. Knocking out *SYISL* alleviates dexamethasone-induced skeletal muscle atrophy. As a sponge for miR-23a-3p, miR-103-3p, and miR-205-5p, SYISL upregulates the expression of FoxO3a, atrogin-1, and murf-1, thereby promoting skeletal muscle atrophy [207]. Lnc-IRS1, acting as the ceRNA of miR-15a, miR-15b-5p, and miR-15c-5p, regulates IRS1 expression and mitigates dexamethasone-induced skeletal muscle atrophy [208]. 

In multiple skeletal muscle atrophy models, the expression of lnc-MAAT, Gm4544, lnc-Myh, Gm49794, Oip5os1, and Gm46085 is reduced [209,210]. LncMAAT negatively regulates miR-29b transcription through a trans-regulatory module involving SOX6, and increases the expression of neighboring gene Mbnl1 through a cis-regulatory module, thereby improving angiotensin-induced skeletal muscle atrophy [209]. Lnc-MAR1 acts as a sponge for microRNA-487b, promoting the expression of wnt5 and improving skeletal muscle atrophy [211]. In tail suspension-treated mouse TA muscles, lnc-MD1, lnc-Myod, and IG-DMR are increased, while Gtl2 expression is reduced. In casting-induced skeletal muscle atrophy, lnc-Myod and linc-MD1 expression increases, while Dum decreases. In cancerous muscle atrophy, Gtl2, DRR, and IG-DMR expression is reduced. In starvation-induced skeletal muscle atrophy, the expression of lnc-Myod, Gtl2, DRR, Dum, H19, and IG-DMR is reduced [212].

In the gastrocnemius muscle of mice with suspension-induced skeletal muscle atrophy, the expression of lnc-MUMA and lnc-H19 is significantly reduced [213,214]. Lnc-MUMA acts as a miR-762 sponge to regulate Myog expression, thereby improving hindlimb suspension-induced skeletal muscle atrophy [213].

Compared to the control group, the expression of Atrolnc-1, lnc-MD1, Myolinc, lnc-MyoD, Dum, and MAR1 significantly increases in starved tissues or cells, while lnc-mg is reduced. Atrolnc-1 expression also increases in the muscles of cancer cachexia and CKD mice. C/EBP-alpha binds to the promoter of Atrolnc-1, promoting its expression. Overexpression of Atrolnc-1 enhances murf-1 expression induced by the NF-κB pathway through ABIN-1. Knocking down Atrolnc-1 improves skeletal muscle atrophy caused by CKD [215]. 

Lnc-Myh is downregulated in ALS and denervation-induced skeletal muscle atrophy, whereas H19, Dancr, Gas5, Igf2os, MiR22hg, Airn, Neat1, Snhg1, and Pvt1 are upregulated [216]. Lnc-mg promotes MuSC differentiation by acting as a competitive endogenous RNA (ceRNA) for microRNA-125b to regulate IGF-2 expression [217]. In denervation-induced skeletal muscle atrophy, Myoparr, linc-MD1, LncMyod, and H19 increase while DRR, DUM, Gtl2, and IG-DMR decrease [212]. Lnc-Myoparr interacts with Ddx17 and modulates the association between histone acetyltransferase PCAF and Ddx17 [218]. Additionally, Myoparr regulates the BMP pathway, and its knockdown ameliorates denervation-induced skeletal muscle atrophy [219].

In the gastrocnemius muscle of db/db mice, the expression of 1700047G03Rik and Gm31814 increases, while Gm20743, Gm35438, Gm36131, and A330074k22rik decrease. In palmitic acid-induced C2C12 cells, the expression patterns of the remaining five lncRNAs mirror those in the gastrocnemius muscle of db/db mice, except for the decreased expression of Gm38141 [220]. 

#### 4.4.3. circRNA

Circular RNA (circRNA) is a type of non-coding RNA molecule characterized by a closed circular structure, lacking 5′ and 3′ ends. This unique structure confers high stability and resistance to exonuclease degradation. CircRNA can be classified into ex-on-circRNA (ecircRNA), exon-intron-circRNA (EIcircRNA), and intron-circRNA (ciRNA). CircRNA serves as a miRNA sponge, regulating the expression, transcription, translation, protein function, and localization of target genes.

CircTmeff1 is highly expressed in various types of skeletal muscle atrophy, including denervation-, mobilization-, dexamethasone-, TNF-α-, and angiotensin II-induced models. Overexpression of circTmeff1 activates the cGAS/STING pathway via TDP-43 and encodes TMEFF1-339aa, leading to skeletal muscle atrophy. Inhibition of circT-meff1 ameliorates skeletal muscle atrophy induced by dexamethasone, denervation, and mobilization [221]. CircCCDC91 adsorbs miR-15a, miR-15b-5p, and miR-15c-5p, activating the IGF-1-PI3K/AKT signaling pathway and regulating IRS1 expression [222].

## 5. Conclusions and Prospects

With the advancement of research, skeletal muscle atrophy has transitioned from a debilitating condition to one that can be delayed or even potentially cured. Currently, skeletal muscle atrophy is primarily managed through exercise, dietary supplements, and drug treatments, but targeted therapies remain elusive. Thus, improving skeletal muscle atrophy remains a significant challenge for clinicians.

Epigenetics has garnered widespread attention and achieved significant progress in recent years. This article summarizes the role of epigenetics in skeletal muscle atrophy. DNA methylation, histone modification, RNA modification, and non-coding RNA are crucial in the progression of skeletal muscle atrophy. However, the study of epigenetics in skeletal muscle atrophy is still in its infancy. Further research on molecular targets is essential, and developing viable treatments for these newly discovered processes and their associated signaling networks is imperative.

## Figures and Tables

**Table 1:** HADCs inhibitors in skeletal muscle atrophy.

Name	Mechanism	Results	Reference
Trichostatin A	Inhibiting HDAC activity	Promoting atrogin-1 mRNA level	Alamdari et al. (2010) [87]
		Improving amyotrophic lateral sclerosis	Yoo et al. (2011) [100]
		Improving unloaded-induced skeletal muscle atrophy	Aucouturier et al. (2015) [101]
	Inhibiting HDAC1/2	Improving muscle atrophy induced by cigarette smoke exposure	Ding et al. (2019) [99]
MS-275	Inhibiting class I HDAC	Improving fasting and denervation-induced skeletal muscle atrophy	Beharry et al. (2014) [97]
Butyrate		Improving sarcopenia	Walsh et al. (2015) [102]
HC toxin		Improving starvation-induced muscle atrophy	Tan et al. (2015) [106]
Valproic acid		Attenuating cancer cachexia- induced skeletal muscle atrophy	Sun et al. (2016) [105]
NVS-HD1	Inhibiting HDAC4	Improving dexamethasone-induced skeletal muscle atrophy	Luo et al. (2019) [95]
LMK-235	Inhibiting HDAC5	Improving unloading-induced muscle atrophy	Mochalova et al. (2020) [103]
Tubastatin A	Inhibiting HDAC6	Improving Duchenne muscular dystrophy	Osseni et al. (2022) [104]
trichostatin A, apicidin, romidepsin	Inhibiting HDAC1/2	attenuating dexamethasone-induced muscle atrophy	Habibian et al. (2023) [107]

## Data Availability

No new data were created or analyzed in this study. Data sharing is not applicable to this article.

## Abbreviations

COPD	chronic obstructive pulmonary disease
ALS	amyotrophic lateral sclerosis
SMA	spinal muscular atrophy
DMD	Duchenne muscular dystrophy
CKD	chronic kidney disease
atrogin-1	muscle atrophy F-box/MAFbx
murf-1	muscle ring finger-1
eIF3-f	eukaryotic translation initiation factor 3 subunit f
MyoD	myogenic differentiation antigen
Mib1	mindbomb-1
UPS	ubiquitin–proteasome system
EDL	extensor digitorum longus
Atg7	autophagy related 7
KO	knockout
Atg5	autophagy related 5
H_2_O_2_	hydrogen peroxide
PI3K	phosphoinositide 3-kinase
AKT	serine/threonine-specific protein kinase
mTOR	mechanistic target of rapamycin kinase
P70S6K	p70 ribosomal protein S6 kinase
4EBP1	e IF4E-binding protein 1
Foxo	forkhead box O
IGF-1	insulin-like growth factor 1
AMPK	AMP-activated protein kinase
mtDNA	mitochondrial DNA
IL-1	interleukin-1
TNF-α	tumor necrosis factor alpha
IL-6	interleukin-6
CRP	C-reactive protein
NF-κB	nuclear factor kappa-light-chain-enhancer of activated B cells
Nfkb1	nuclear factor kappa B subunit 1
GDF-8	growth differentiation factor-8
TGFβ	transforming growth factor β
5mC	5-methylcytosine
DNMT	DNA methyltransferase
5hmC	5-hydroxymethylcytosine
5caC	5-carboxylcytosine
5fC	5-formylcytosine
KEGG	kyoto encyclopedia of genes and genomes
DMR	differentially methylated region
DNMT	DNA methyltransferases
TET	ten-eleven translocation
HATs	histone acetyltransferases
HDACs	histone deacetylases
MLC3	myosin light chains 3
HMT	histone methyltransferase
me1	monomethylation
me2	dimethylation
me3	trimethylation
PRMT	protein arginine methyltransferases
H2AX	H2A histone family member X
m6A	N6 adenylate methylation
METTL	methyltransferase like
FTO	fat mass and obesity-associated
ALKBH5	alpha-ketoglutarate-dependent dioxygenase alkB homolog 5
DAA	3-Dezidenosine
R-2HG	R-2-HydroxyglutaTa
s2U	2-thiouridine
Ψ	pseudouridine
ADARs	adenosine deaminases acting on RNA
ncRNA	noncoding RNA
lncRNA	long noncoding RNA
miRNA	microRNA
3′UTR	3′ untranslated region
siRNA	small interfering RNA
RNAi	RNA interference
piRNA	PIWI-interacting RNA
snoRNA	small nucleolar RNA
OSCC	oral squamous cell carcinoma
ceRNA	competing endogenous RNAs
